# Supplementary material for: A foundation model for human-AI collaboration in medical literature mining
Source: Nat Commun. 2025 Sep 24;16:8361. doi: 10.1038/s41467-025-62058-5 (PMC12460617; doi:10.1038/s41467-025-62058-5)
Supplement: Supplementary file 1 — Supplementary information [file 41467_2025_62058_MOESM1_ESM.pdf]

| PMID | LEADS | GPT-4o | Deep Research | Difference |
|---|---|---|---|---|
| 39976227 | 0.50 | 0.00 | 1.00 | -0.50 |
| 39981752 | 1.00 | 0.00 | 0.50 | 0.50 |
| 40008607 | 0.70 | 0.00 | 0.60 | 0.10 |
| 40013543 | 1.00 | 0.00 | 0.50 | 0.50 |
| 40028776 | 0.44 | 0.50 | 0.33 | -0.06 |
| 39775922 | 0.03 | 0.31 | - | -0.28 |
| 39777725 | 0.00 | 0.00 | - | 0.00 |
| 39791480 | 0.50 | 0.00 | - | 0.50 |
| 39799985 | 0.57 | 0.00 | - | 0.57 |
| 39803834 | 0.36 | 0.00 | - | 0.36 |
| 39807668 | 0.46 | 0.00 | - | 0.46 |
| 39817620 | 0.14 | 0.00 | - | 0.14 |
| 39820897 | 0.30 | 0.00 | - | 0.30 |
| 39822092 | 1.00 | 0.00 | - | 1.00 |
| 39831540 | 0.83 | 0.19 | - | 0.64 |
| 39868562 | 0.78 | 0.73 | - | 0.06 |
| 39868569 | 0.62 | 0.01 | - | 0.61 |
| 39873294 | 0.64 | 0.00 | - | 0.64 |
| 39878152 | 0.38 | 0.08 | - | 0.29 |
| 39878158 | 0.05 | 0.07 | - | -0.02 |
| 39880377 | 0.71 | 0.03 | - | 0.68 |
| 39912435 | 1.00 | 0.50 | - | 0.50 |
| 39912443 | 0.63 | 0.05 | - | 0.58 |
| 39927511 | 0.00 | 0.00 | - | 0.00 |
| 39927555 | 1.00 | 0.00 | - | 1.00 |
| 39932103 | 0.02 | 0.42 | - | -0.40 |
| 39945386 | 0.59 | 0.00 | - | 0.59 |
| 39963952 | 0.00 | 0.04 | - | -0.04 |
| 39963955 | 0.56 | 0.11 | - | 0.44 |
| 39968829 | 1.00 | 1.00 | - | 0.00 |
| 39968844 | 0.00 | 0.00 | - | 0.00 |
| Average | 0.51 | 0.13 | | 0.30 |

Extended Fig. 1: A pseudo-prospective evaluation for the study search performance of the selected methods based on 31 systematic reviews published after 2025. For each review, we computed Recall@2000, with the 'Difference' column representing the performance gap between LEADS and the best baseline for that review. Green highlights cases where LEADS performed at least as well as the best baseline. 'Deep Research' was conducted by submitting the research question to ChatGPT with Deep Research mode enabled.

| PMID | LEADS_recall_50 | GPT4o_recall_50 | Difference |
|---|---|---|---|
| 39775922 | 1.00 | 1.00 | 0.00 |
| 39777725 | 1.00 | 1.00 | 0.00 |
| 39791480 | 1.00 | 1.00 | 0.00 |
| 39799985 | 0.43 | 0.47 | -0.04 |
| 39803834 | 0.70 | 0.82 | -0.12 |
| 39807668 | 1.00 | 0.97 | 0.03 |
| 39817620 | 1.00 | 1.00 | 0.00 |
| 39820897 | 1.00 | 0.95 | 0.05 |
| 39822092 | 1.00 | 1.00 | 0.00 |
| 39831540 | 0.94 | 0.94 | 0.00 |
| 39868562 | 0.84 | 0.92 | -0.08 |
| 39868569 | 0.35 | 0.39 | -0.04 |
| 39873294 | 1.00 | 1.00 | 0.00 |
| 39878152 | 0.57 | 0.61 | -0.04 |
| 39878158 | 0.35 | 0.38 | -0.02 |
| 39880377 | 1.00 | 1.00 | 0.00 |
| 39912435 | 1.00 | 1.00 | 0.00 |
| 39912443 | 0.77 | 0.81 | -0.05 |
| 39927511 | 0.82 | 0.82 | 0.00 |
| 39927555 | 1.00 | 1.00 | 0.00 |
| 39932103 | 0.28 | 0.31 | -0.03 |
| 39945386 | 0.72 | 0.70 | 0.02 |
| 39963952 | 0.61 | 0.61 | 0.00 |
| 39963955 | 1.00 | 1.00 | 0.00 |
| 39968829 | 1.00 | 1.00 | 0.00 |
| 39968844 | 1.00 | 1.00 | 0.00 |
| 39976227 | 1.00 | 1.00 | 0.00 |
| 39981752 | 1.00 | 1.00 | 0.00 |
| 40008607 | 0.90 | 0.90 | 0.00 |
| 40013543 | 1.00 | 1.00 | 0.00 |
| 40028776 | 1.00 | 1.00 | 0.00 |
| Average | 0.85 | 0.86 | -0.01 |

Extended Fig. 2: A pseudo-prospective evaluation of study screening performance using 31 systematic reviews published after 2025. For each review, we computed Recall@50, with the 'Difference' column indicating the performance gap between LEADS and GPT-4o. Green highlights cases where LEADS performed at least as well as GPT-4o.

Health

| Method | P@10 | P@20 | P@50 |
|---|---|---|---|
| Dense | 0.205 | 0.195 | 0.141 |
| Mistral-7B | 0.323 | 0.247 | 0.164 |
| Haiku-3 | 0.318 | 0.265 | 0.173 |
| GPT-3.5 | 0.293 | 0.251 | 0.171 |
| GPT-4o | 0.371 | 0.297 | 0.183 |
| LEADS | 0.371 | 0.304 | 0.193 |
| Δ vs. best | +0.000 | +0.007 | +0.010 |

Neoplasms

| Method | P@10 | P@20 | P@50 |
|---|---|---|---|
| Dense | 0.205 | 0.147 | 0.081 |
| Mistral-7B | 0.216 | 0.176 | 0.096 |
| Haiku-3 | 0.258 | 0.195 | 0.107 |
| GPT-3.5 | 0.247 | 0.179 | 0.101 |
| GPT-4o | 0.316 | 0.213 | 0.118 |
| LEADS | 0.279 | 0.184 | 0.111 |
| Δ vs. best | -0.037 | -0.029 | -0.007 |

Metabolic

| Method | P@10 | P@20 | P@50 |
|---|---|---|---|
| Dense | 0.214 | 0.157 | 0.1 |
| Mistral-7B | 0.25 | 0.186 | 0.106 |
| Haiku-3 | 0.3 | 0.243 | 0.131 |
| GPT-3.5 | 0.271 | 0.204 | 0.124 |
| GPT-4o | 0.314 | 0.225 | 0.119 |
| LEADS | 0.357 | 0.232 | 0.127 |
| Δ vs. best | +0.043 | -0.011 | -0.004 |

Musculoskeletal

| Method | P@10 | P@20 | P@50 |
|---|---|---|---|
| Dense | 0.232 | 0.224 | 0.155 |
| Mistral-7B | 0.272 | 0.230 | 0.152 |
| Haiku-3 | 0.340 | 0.298 | 0.193 |
| GPT-3.5 | 0.288 | 0.224 | 0.143 |
| GPT-4o | 0.344 | 0.286 | 0.196 |
| LEADS | 0.408 | 0.310 | 0.183 |
| Δ vs. best | +0.064 | +0.012 | -0.013 |

Mental

| Method | P@10 | P@20 | P@50 |
|---|---|---|---|
| Dense | 0.219 | 0.206 | 0.135 |
| Mistral-7B | 0.294 | 0.238 | 0.149 |
| Haiku-3 | 0.362 | 0.291 | 0.174 |
| GPT-3.5 | 0.269 | 0.238 | 0.15 |
| GPT-4o | 0.375 | 0.306 | 0.176 |
| LEADS | 0.388 | 0.297 | 0.168 |
| Δ vs. best | +0.013 | -0.009 | -0.008 |

Digestive

| Method | P@10 | P@20 | P@50 |
|---|---|---|---|
| Dense | 0.242 | 0.258 | 0.197 |
| Mistral-7B | 0.225 | 0.204 | 0.172 |
| Haiku-3 | 0.367 | 0.337 | 0.222 |
| GPT-3.5 | 0.35 | 0.283 | 0.197 |
| GPT-4o | 0.367 | 0.329 | 0.215 |
| LEADS | 0.367 | 0.337 | 0.228 |
| Δ vs. best | **+0.000** | **+0.000** | **+0.006** |

Circulatory

| Method | P@10 | P@20 | P@50 |
|---|---|---|---|
| Dense | 0.195 | 0.148 | 0.080 |
| Mistral-7B | 0.224 | 0.160 | 0.078 |
| Haiku-3 | 0.271 | 0.190 | 0.101 |
| GPT-3.5 | 0.200 | 0.150 | 0.085 |
| GPT-4o | 0.262 | 0.195 | 0.102 |
| LEADS | 0.295 | 0.195 | 0.097 |
| Δ vs. best | +0.024 | +0.000 | -0.005 |

Infectious

| Method | P@10 | P@20 | P@50 |
|---|---|---|---|
| Dense | 0.2 | 0.193 | 0.131 |
| Mistral-7B | 0.267 | 0.223 | 0.145 |
| Haiku-3 | 0.3 | 0.25 | 0.151 |
| GPT-3.5 | 0.233 | 0.19 | 0.137 |
| GPT-4o | 0.347 | 0.28 | 0.153 |
| LEADS | 0.327 | 0.27 | 0.155 |
| Δ vs. best | -0.020 | -0.010 | +0.002 |

Nervous

| Method | P@10 | P@20 | P@50 |
|---|---|---|---|
| Dense | 0.242 | 0.258 | 0.197 |
| Mistral-7B | 0.225 | 0.204 | 0.172 |
| Haiku-3 | 0.367 | 0.337 | 0.222 |
| GPT-3.5 | 0.35 | 0.283 | 0.197 |
| GPT-4o | 0.367 | 0.329 | 0.215 |
| LEADS | 0.367 | 0.337 | 0.228 |
| Δ vs. best | **+0.000** | **+0.000** | **+0.006** |

Extended Fig. 3: Study screening performance across nine medical topics. Precision at top-10 (P@10), top-20 (P@20), and top-50 (P@50) is reported for LEADS and baseline methods, including dense retrieval and large language models (Mistral-7B, Haiku-3, GPT-3.5, GPT-4o). The last row in each topic (Δ vs. best) shows LEADS' difference from the best baseline. Green cells indicate improvement, while red cells indicate underperformance.

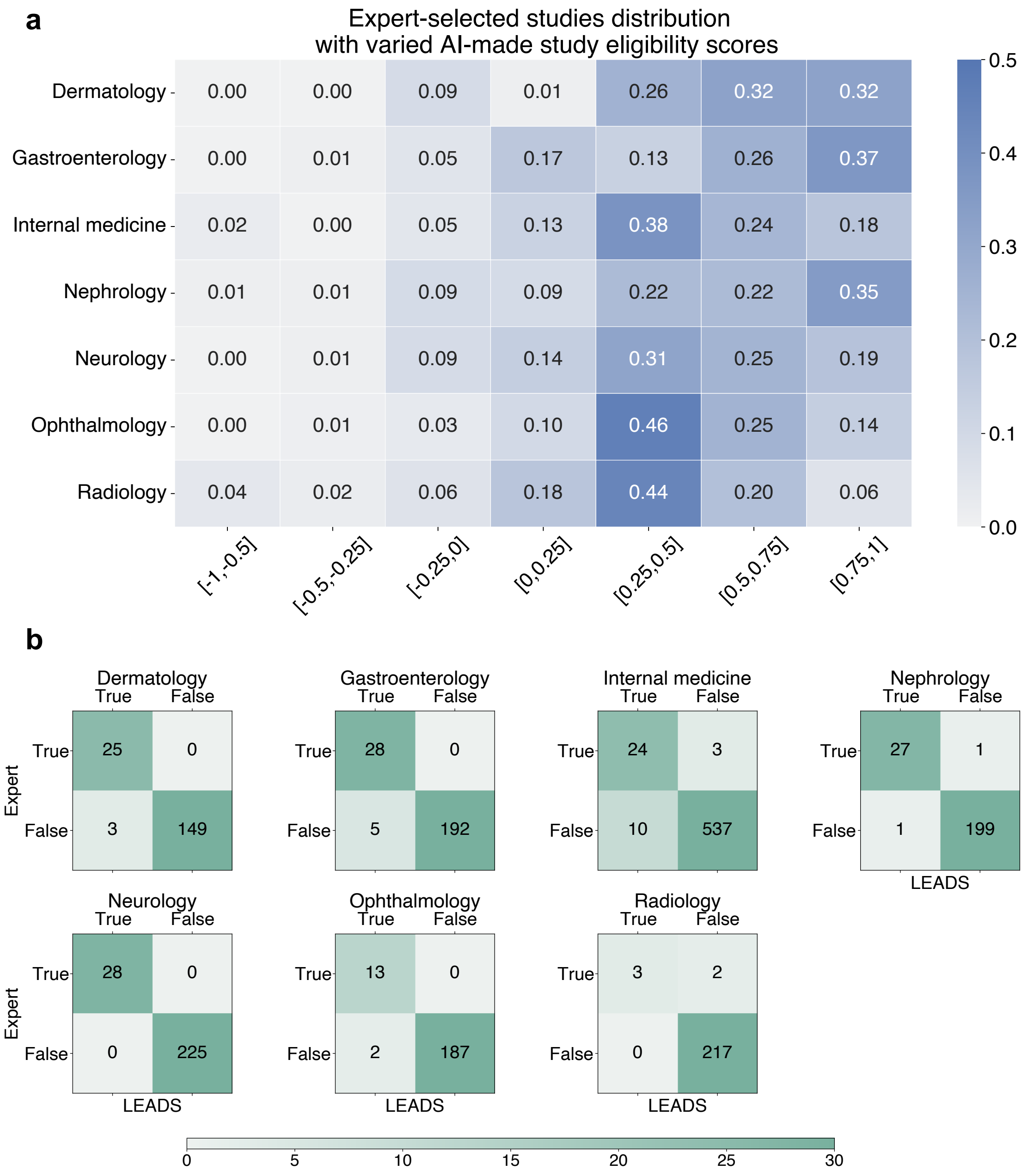


Extended Fig. 4: **The distribution of expert-selected studies and AI-generated eligibility assessments. a**, studies are stratified by the overall eligibility scores assigned by LEADS, with the percentage of selected studies calculated for each group (row percentages sum to one). **b**, the confusion matrix compares LEADS-generated eligibility assessments and expert decisions, treating score group [0.75, 1] as LEADS-predicted "True" and [-1, -0.5] as LEADS-predicted "False."

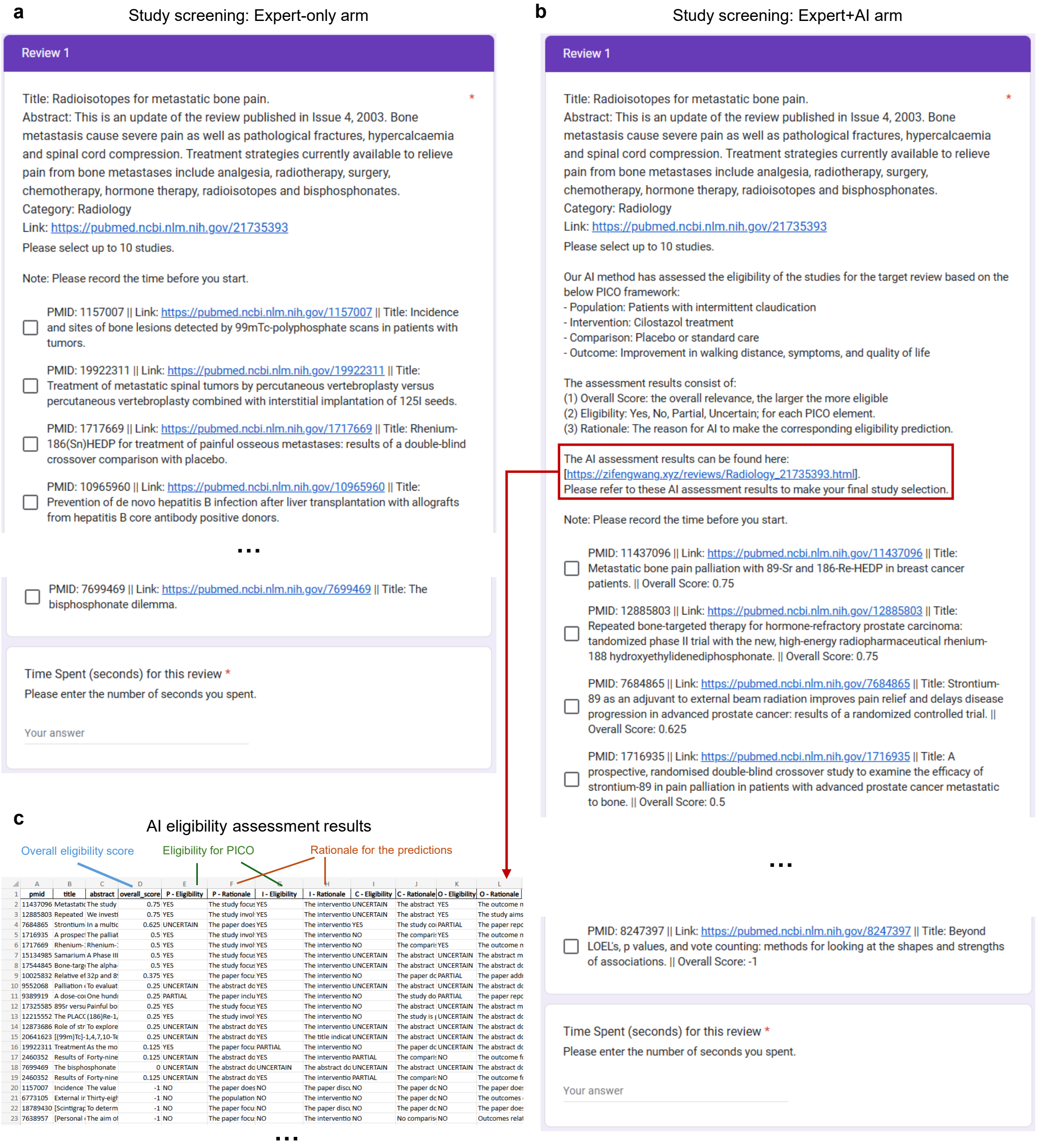


Extended Fig. 5: **The forms shared with experts to complete the pilot user study for study screening.** **a**, the Expert-only arm where one needs to find eligible studies from a randomly shuffled list of candidates and submit the results with the time spent. **b**, the Expert+AI arm where one needs to find eligible studies referring to the AI eligibility assessment results. **c**, the AI assessment results that participants read when making the decisions. The studies are ranked by the overall scores, with the predictions and rationale breaking down for each PICO element.

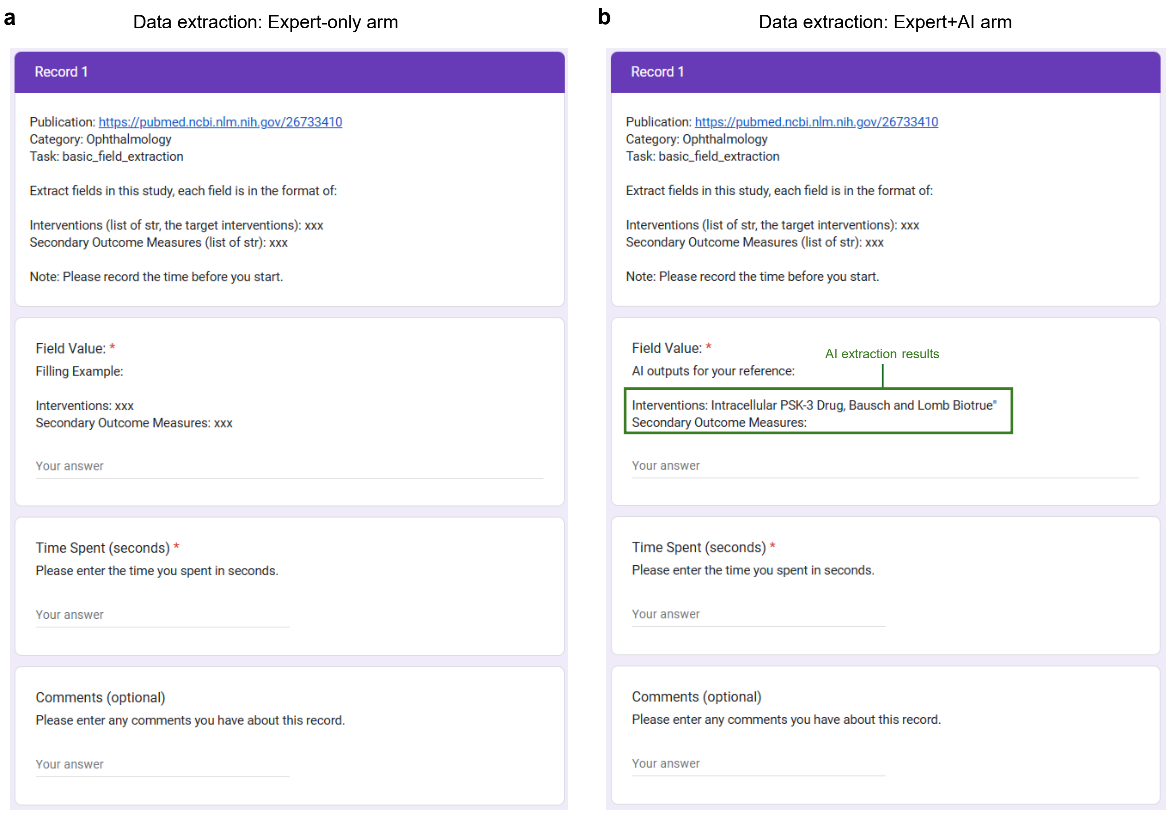


Extended Fig. 6: **The forms shared with experts to complete the pilot user study for data extraction.** **a**, the Expert-only arm where one needs to follow the definition of the target field and extract the results from the raw study document. **b**, the Expert+AI arm, where one can refer to AI extraction results to extract the target field values from the raw study document.

a

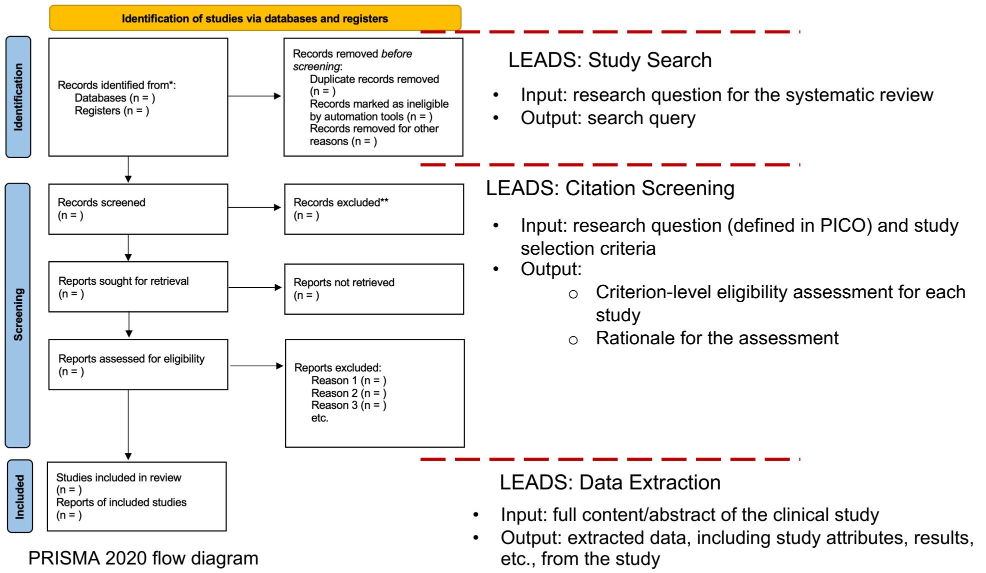


PRISMA 2020 flow diagram
Source: *Page MJ, et al. BMJ 2021;372:n71. doi: 10.1136/bmj.n71.*

b

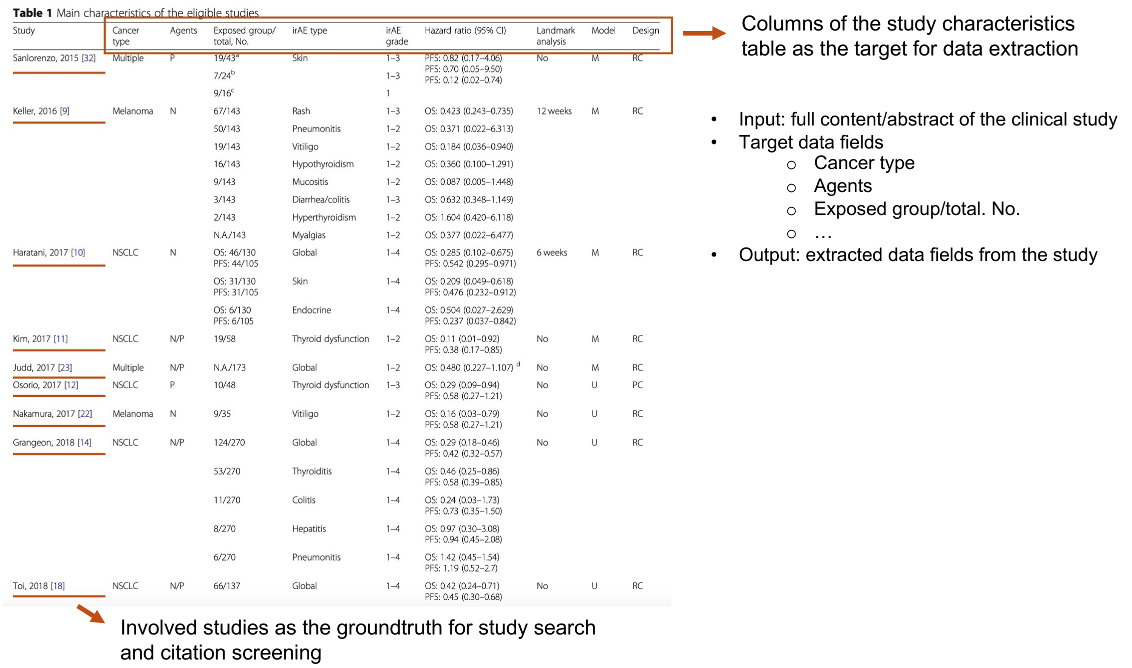

**Table 1** Main characteristics of the eligible studies

| Study | Cancer type | Agents | Exposed group/ total, No. | irAE type | irAE grade | Hazard ratio (95% CI) | Landmark analysis | Model | Design |
|---|---|---|---|---|---|---|---|---|---|
| Sanlorenzo, 2015 [32] | Multiple | P | 19/43[a] | Skin | 1–3 | PFS: 0.82 (0.17–4.06) | No | M | RC |
| | | | 7/24[b] | | 1–3 | PFS: 0.70 (0.05–9.50) | | | |
| | | | 9/16[c] | | 1 | PFS: 0.12 (0.02–0.74) | | | |
| Keller, 2016 [9] | Melanoma | N | 67/143 | Rash | 1–3 | OS: 0.423 (0.243–0.735) | 12 weeks | M | RC |
| | | | 50/143 | Pneumonitis | 1–2 | OS: 0.371 (0.022–6.313) | | | |
| | | | 19/143 | Vitiligo | 1–2 | OS: 0.184 (0.036–0.940) | | | |
| | | | 16/143 | Hypothyroidism | 1–2 | OS: 0.360 (0.100–1.291) | | | |
| | | | 9/143 | Mucositis | 1–2 | OS: 0.087 (0.005–1.448) | | | |
| | | | 3/143 | Diarrhea/colitis | 1–3 | OS: 0.632 (0.348–1.149) | | | |
| | | | 2/143 | Hyperthyroidism | 1–2 | OS: 1.604 (0.420–6.118) | | | |
| | | | N.A./143 | Myalgias | 1–2 | OS: 0.377 (0.022–6.477) | | | |
| Haratani, 2017 [10] | NSCLC | N | OS: 46/130<br>PFS: 44/105 | Global | 1–4 | OS: 0.285 (0.102–0.675)<br>PFS: 0.542 (0.295–0.971) | 6 weeks | M | RC |
| | | | OS: 31/130<br>PFS: 31/105 | Skin | 1–4 | OS: 0.209 (0.049–0.618)<br>PFS: 0.476 (0.232–0.912) | | | |
| | | | OS: 6/130<br>PFS: 6/105 | Endocrine | 1–4 | OS: 0.504 (0.027–2.629)<br>PFS: 0.237 (0.037–0.842) | | | |
| Kim, 2017 [11] | NSCLC | N/P | 19/58 | Thyroid dysfunction | 1–2 | OS: 0.11 (0.01–0.92)<br>PFS: 0.38 (0.17–0.85) | No | M | RC |
| Judd, 2017 [23] | Multiple | N/P | N.A./173 | Global | 1–2 | OS: 0.480 (0.227–1.107) [d] | No | M | RC |
| Osorio, 2017 [12] | NSCLC | P | 10/48 | Thyroid dysfunction | 1–3 | OS: 0.29 (0.09–0.94)<br>PFS: 0.58 (0.27–1.21) | No | U | PC |
| Nakamura, 2017 [22] | Melanoma | N | 9/35 | Vitiligo | 1–2 | OS: 0.16 (0.03–0.79)<br>PFS: 0.58 (0.27–1.21) | No | U | RC |
| Grangeon, 2018 [14] | NSCLC | N/P | 124/270 | Global | 1–4 | OS: 0.29 (0.18–0.46)<br>PFS: 0.42 (0.32–0.57) | No | U | RC |
| | | | 53/270 | Thyroiditis | 1–4 | OS: 0.46 (0.25–0.86)<br>PFS: 0.58 (0.39–0.85) | | | |
| | | | 11/270 | Colitis | 1–4 | OS: 0.24 (0.03–1.73)<br>PFS: 0.73 (0.35–1.50) | | | |
| | | | 8/270 | Hepatitis | 1–4 | OS: 0.97 (0.30–3.08)<br>PFS: 0.94 (0.45–2.08) | | | |
| | | | 6/270 | Pneumonitis | 1–4 | OS: 1.42 (0.45–1.54)<br>PFS: 1.19 (0.52–2.7) | | | |
| Toi, 2018 [18] | NSCLC | N/P | 66/137 | Global | 1–4 | OS: 0.42 (0.24–0.71)<br>PFS: 0.45 (0.30–0.68) | No | U | RC |



Extended Fig. 7: LEADS aligns with PRISMA's systematic review process by streamlining key steps: (1) Identification, generating search terms from PICO elements for comprehensive study retrieval; (2) Screening, creating and applying inclusion/exclusion criteria to assess study eligibility, and (3) Inclusion, extracting target data fields, providing sources, and aggregating study outcomes via meta-analysis. This design enables seamless integration into the systematic literature review workflow while supporting human-AI collaboration. **b**, LEADS is developed and evaluated using published systematic reviews. Given a review paper, the included studies are extracted as target studies for identification and screening, while target data from the study characteristic table serves as ground truth for data extraction. This setup ensures evaluation accuracy and alignment with PRISMA practices.

***Study characteristic extraction: Input***

```
# CONTEXT #
You are tasked with analyzing clinical trial study reports or papers to extract specific information as structured data.

# OBJECTIVE #
The user will provide a list of fields they are interested in, along with a natural language description for each field to guide you on what content to look for and from which parts of the report to extract it.

# PAPER CONTENT #
Screen-and-treat approaches for cervical cancer prevention in low-resource settings: a randomized controlled trial. Non-cytology-based screen-and-treat approaches for cervical cancer prevention have been developed for low-resource settings, but few have directly addressed efficacy. […] with delayed evaluation at both 6 and 12 months. Trial Registration http://clinicaltrials.gov Identifier: NCT00233727.

# TARGET #
- Number of fields: 4
- Field definition: <field id=1>"Conditions, list of str: the target conditions of the study"</field>
<field id=2>"Interventions, list of str: the target interventions"</field>
<field id=3>"Primary Outcome Measures, str"</field>
<field id=4>"Secondary Outcome Measures, list of str"</field>

# RESPONSE #
A syntactically correct JSON string representing a list of dictionary with two keys: name and value.
Format:
```json
[
   {
      "name":  \ str
      "value":  \ str
   },
   {
      "name":, \ str
      "value":  \ str
   },
   ...
]
```
```

***Study characteristic extraction: Output***

```
[
   {
      "name": "Conditions",
      "value": "Cervical Cancer"
   },
   {
      "name": "Interventions",
      "value": "HPV DNA Testing + Cryosurgery, VIA + Cryosurgery"
   },
   {
      "name": "Primary Outcome Measures",
      "value": "Efficacy of \"Screen and Treat\" + Cryosurgery"
   },
   {
      "name": "Secondary Outcome Measures",
      "value": "HIV incidence, Safety of Cryosurgery"
   }
]
```

Extended Fig. 8: Example inputs and outputs for the study characteristics extraction task.

## ***Arm design extraction: Input***

(TITLE) Vaccination response to tetanus toxoid and 23-valent pneumococcal vaccines following administration of a single dose of abatacept: a randomized, open-label, parallel group study in healthy subjects
(ABSTRACT) The effect of abatacept, a selective T-cell co-stimulation modulator, on vaccination has not been previously investigated. In this open-label, single-dose, randomized, parallel-group, controlled study, the effect of a single 750 mg infusion of abatacept on the antibody response to the intramuscular tetanus toxoid vaccine (primarily a memory response to a T-cell-dependent peptide antigen) and the intramuscular 23-valent pneumococcal vaccine (a less T-cell-dependent response to a polysaccharide antigen) was measured in 80 normal healthy volunteers. Subjects were uniformly randomized to receive one of four treatments: Group A (control group), subjects received vaccines on day 1 only; Group B, subjects received vaccines 2 weeks before abatacept; Group C, subjects received vaccines 2 weeks after abatacept; and Group D, subjects received vaccines 8 weeks after abatacept. Anti-tetanus and anti-pneumococcal (Danish serotypes 2, 6B, 8, 9V, 14, 19F and 23F) antibody titers were measured 14 and 28 days after vaccination. While there were no statistically significant differences between the dosing groups, geometric mean titers following tetanus or pneumococcal vaccination were generally lower in subjects who were vaccinated 2 weeks after receiving abatacept, compared with control subjects. A positive response (defined as a twofold increase in antibody titer from baseline) to tetanus vaccination at 28 days was seen, however, in â¥ 60% of subjects across all treatment groups versus 75% of control subjects. Similarly, over 70% of abatacept-treated subjects versus all control subjects (100%) responded to at least three pneumococcal serotypes, and approximately 25â30% of abatacept-treated subjects versus 45% of control subjects responded to at least six serotypes.
(INTRO) Introduction
(INTRO) Treatment with abatacept has demonstrated efficacy in patients with active rheumatoid arthritis (RA) and an inadequate response to methotrexate, and in those with an inadequate response to anti-TNF therapy. Abatacept is a soluble fusion protein consisting of the extracellular domain of human cytotoxic T-lymphocyte-associated antigen-4 linked to the Fc (hinge, CH2 and CH3 domains) portion of human IgG1, which has been modified
[... Omitted to avoid clutter]
(DISCUSS) Abatacept prevents the activation of naive T cells by inhibiting the second signal required for their co-stimulation. This signal is mediated by CD80 and CD86, which is expressed on antigen-presenting cells, and by CD28, which is expressed on T cells. Abatacept may also reduce the activation of memory T cells (although to a lesser extent than for naÃ¯ve T cells). This is consistent with a reduced response against tetanus toxoid. The inhibition of the CD80/CD86:CD28 co-stimulatory signal may also potentially prevent the T-cell 'help' needed for optimal differentiation of CD80/CD86-expressing B cells into plasma cells, which ultimately secrete antibodies. This inhibition of B cellâT cell help may be a reason for the reduced antibody response to thymus-independent polysaccharide antigens such as those contained in the pneumococcal vaccine â responses that cannot be considered completely T-cell independent since they are enhanced by T-cell help. Finally, since abatacept inhibits one of several mediators of co-stimulation, the partial inhibition observed here is likely to reflect the redundancy of the co-stimulation mechanism.
(DISCUSS) This study analyzed the response in healthy volunteers with a normal immune system to a single dose of abatacept. Future studies are needed to determine the optimal timing of vaccination in RA patients receiving abatacept continuously, possibly with other concomitant disease-modifying antirheumatic drugs such as methotrexate.
(CONCL) Conclusion
(CONCL) This study suggests that abatacept blunts the effectiveness of the immune response, but does not significantly inhibit the ability of healthy subjects to develop a clinically significant or positive immune response (at least a twofold increase above baseline) to both tetanus toxoid and 23-valent pneumococcal vaccines.

## ***Arm design extraction: Output***


```
[
    {
        "label": "Group 1",
        "type": "EXPERIMENTAL",
        "interventionNames": [
            "Drug: Tetanus + pnemococcal vaccines alone"
        ]
    },
    {
        "label": "Group 2",
        "type": "ACTIVE_COMPARATOR",
        "interventionNames": [
            "Drug: Abatacept + vaccines"
        ]
    },
    {
        "label": "Group 3",
        "type": "ACTIVE_COMPARATOR",
        "interventionNames": [
            "Drug: Abatacept + vaccines"
        ]
    },
    {
        "label": "Group 4",
        "type": "ACTIVE_COMPARATOR",
        "interventionNames": [
            "Drug: Abatacept + Vaccines"
        ]
    }
]
```


Extended Fig. 9: Example inputs and outputs for the arm design extraction task.

***Participant statistics extraction: Input***

~~~~
# CONTEXT #
You are tasked with analyzing clinical trial study reports or papers to extract specific information as structured data.

# PAPER CONTENT #
(TITLE) Protocol for the Locomotor Experience Applied Post-stroke (LEAPS) trial: a randomized controlled trial
(ABSTRACT) Background
(ABSTRACT) Locomotor training using body weight support and a treadmill as a therapeutic modality for rehabilitation of walking post-stroke is being
[... Omitted to avoid clutter]
(DISCUSS) Current study status: Enrolling Patients

# TARGET #
Given the following information:
Parameter Type: MEAN
Unit of Measurement: days
Participant Groups Definition: [{'groupId': 'BG000', 'value': 139.0, 'unit': 'Participants', 'def': 'Early Locomotor Training Program: Stepping on a treadmill
with partial body weight support and manual assistance as needed for 20-30 minutes at 2.0 mph, followed by a progressive overground walking program
for 20 minutes delivered at 2 months post-stroke.'}, [… more group definitions], {'groupId': 'BG003', 'value': 408.0, 'unit': 'Participants', 'def': 'Total: Total
of all reporting groups'}]

Where:
"groupId" is the group identifier
"unit" is the unit of measurement for the group
"value" is the numerical value representing the group's characteristic
"def" is the definition or description of the group

Extract the target participant's characteristics about Participants characteristics: Time since stroke to randomization - days.

# RESPONSE #
A syntactically correct JSON string:

Format:
```json
{
   "results": [ \ the list of characteristics for the groups
      {
         "groupId": \ str
         "value": \ float or int, the value for the group
         "note": \ str, the note for the value
      },
      ... \ more results
   ]
}
```
~~~~

***Participant statistics extraction: Output***


```
{
   "results": [
      {
         "groupId": "BG000",
         "value": 64.1,
         "note": ""
      },
      {
         "groupId": "BG001",
         "value": 64.18,
         "note": ""
      },
      {
         "groupId": "BG002",
         "value": 62.9,
         "note": ""
      },
      {
         "groupId": "BG003",
         "value": 63.8,
         "note": ""
      }
   ]
}
```


Extended Fig. 10: Example inputs and outputs for the participant statistics extraction task.

***Trial result extraction: Input***

````
# CONTEXT #
You are tasked with analyzing clinical trial study reports or papers to extract specific information as structured data.

# PAPER CONTENT #
(TITLE) Comparison of flow characteristics and vascular reactivity of radial artery and long saphenous vein grafts [NCT00139399]
(ABSTRACT) Background
(ABSTRACT) The morphological and functional differences between arteries and veins may have implications on coronary artery bypass graft (CABG)
survival. Although subjective differences have been observed between radial artery (RA) and long saphenous venous (LSV) grafts, these have not been
quantified. This study assessed and compared the flow characteristics and in-vivo graft flow responses of RA and LSV aorto-coronary grafts.
(ABSTRACT) Methods
(ABSTRACT) Angiograms from 52 males taken 3.7 Â± 1.0 months after CABG surgery were analyzed using adjusted Thrombolysis in Myocardial
Infarction (TIMI) frame count. Graft and target coronary artery dimensions were measured using quantitative coronary angiography. Estimated TIMI
velocity (VE) and volume flow (FE) were then calculated. A further 7 patients underwent in-vivo graft flow responses assessments to adenosine,
acetylcholine and isosorbide dinitrate (ISDN) using intravascular Doppler.
(ABSTRACT) Results
(ABSTRACT) The VE for RA grafts was significantly greater than LSV grafts (P = 0.002), however there was no difference in volume FE (P = 0.20). RA
grafts showed positive endothelium-dependent and -independent vasodilatation, and LSV grafts showed no statistically significant response to
adenosine and acetylcholine. There was no difference in flow velocity or volume responses. Seven RA grafts (11%) had compromised patency (4 (6%)
[… Omitted to avoid clutter]
(CONCL) Conclusion

# TARGET #
Extract the results related to the specified outcome and group as follows:

Outcome: Patency Rates, Angiographic patency rates of radial artery and long saphenous vein grafts at follow-up angiography

Group: Saphenous Vein:
Saphenous vein aortocoronary bypass graft

Patients were randomized to receive a long saphenous vein graft to the left circumflex coronary artery territory during CABG surgery

# RESPONSE #
A syntactically correct JSON string:

Format:
```json
{
    "paramType": \ str, the type of the parameters
    "unitOfMeasure": \ str, the unit of the result values
    "timeFrame": \ str, the timeframe
    "unitOfDenom": \ str, the unit of the denomintor for this group
    "denomValue": \ int, the value of the group's denominator
    "results": [ \ list of result values
        {
            "value": int or float \ the result value
            "title": str \ the title for this value
        },... \ more results, if applicable
    ]
}
```
````

***Trial result extraction: Output***


```
{
    "paramType": "COUNT_OF_PARTICIPANTS",
    "unitOfMeasure": "Participants",
    "timeFrame": "5 years",
    "unitOfDenom": "Participants",
    "denomValue": 44.0,
    "results": [
        {
            "value": 38.0,
            "title": "Patent graft"
        },
        {
            "value": 6.0,
            "title": "Occluded graft"
        }
    ]
}
```


Extended Fig. 11: Example inputs and outputs for the trial results extraction task.

***Prompt for Search Query Generation***

```
# CONTEXT #
You are a clinical specialist. You are conducting research and doing a medical literature review.

# OBJECTIVE #
Your task is to create query terms for a search URL to find relevant literature on PubMed or ClinicalTrials.gov.

# RESEARCH DETAILS #
The research is defined by the following PICO elements:
P (Patient, Problem or Population): {P}
I (Intervention): {I}
C (Comparison): {C}
O (Outcome): {O}

# RESPONSE #
Your output should be in the following JSON format:
{{
   "query": "...."
}}
```

Extended Fig. 12: Prompt for the task of search query generation in LEADS. Blue text indicates placeholders for variables within the prompt.

***Prompt for Study Eligibility Prediction***

```
# CONTEXT #
You are a clinical specialist tasked with assessing research papers for inclusion in a systematic literature review based on specific
eligibility criteria.

# OBJECTIVE #
Evaluate each criterion of a given paper to determine its eligibility for inclusion in the review. Provide a list of decisions ("YES",
"PARTIAL", "NO", or "UNCERTAIN") for each eligibility criterion. You must deliver exactly {num_criteria} responses.
1. YES: Meets the criteria.
2. PARTIAL: Partially meets the criteria but not completely.
3. NO: Does not meet the criteria.
4. UNCERTAIN: Uncertain if it meets the criteria.

# IMPORTANT NOTE #
If the information within the provided paper content is insufficient to conclusively evaluate a criterion, you must opt for "UNCERTAIN"
as your response. Avoid making assumptions or extrapolating beyond the provided data, as accurate and reliable responses are crucial,
and fabricating information (hallucinations) could lead to serious errors in the systematic review.
If the information is not applicable N/A, you also must opt for "UNCERTAIN".
Use "PARTIAL" when the paper meets some aspects of the criterion but not all; ensure that the partial fulfillment is based on the
provided data and not on assumptions or incomplete information.

# PAPER DETAILS #
- Provided Paper: {paper_content}

# EVALUATION CRITERIA #
- Number of Criteria: {num_criteria}
- Criteria for Inclusion: {criteria_text}

# RESPONSE #
You are required to output a JSON object containing a list of decisions for each of the {num_criteria} eligibility criteria. Each decision
should directly correspond to one of the criteria and be listed in the order they are presented. Ensure to use "UNCERTAIN" wherever
the paper does not explicitly support a "YES", "PARTIAL", or "NO" decision.
The length of "evaluation" should be exactly {num_criteria}.
For example:
```json
{{
  "evaluations": [ \\ list of eligibility decisions for each criterion
    {{
      "eligibility": "YES", \\ decision for the first criterion
      "rationale": "..." \\ rationale for the decision, limited in 50 tokens
    }},
    {{
      "eligibility": "PARTIAL", \\ decision for the second criterion
      "rationale": "..." \\ rationale for the decision, limited in 50 tokens
    }},
    {{
      "eligibility": "NO", \\ decision for the third criterion
      "rationale": "..." \\ rationale for the decision, limited in 50 tokens
    }},
    {{
      "eligibility": "UNCERTAIN", \\ decision for the fourth criterion
      "rationale": "..." \\ rationale for the decision, limited in 50 tokens
    }},
    ... \\ continue for all criteria
  ]
}}
```
```

Extended Fig. 13: Prompt for the task of study eligibility prediction in LEADS. Blue text indicates placeholders for variables within the prompt.

***Prompt for Study Characteristic Extraction***

```
# CONTEXT #
You are tasked with analyzing clinical trial study reports or papers to extract specific information as structured data.

# OBJECTIVE #
The user will provide a list of fields they are interested in, along with a natural language description for each field to guide you on what content to look for and from which parts of the report to extract it.

# PAPER CONTENT #
{paper_content}

# TARGET #
- Number of fields: {num_field}
- Field definition: {fields_info}

# RESPONSE #
A syntactically correct JSON string representing a list of dictionary with two keys: name and value.
The length of the list should be equal to the number of fields requested.
Format:
```json
[
  {{
    "name": \\ str
    "value": \\ str
  }},
  {{
    "name":, \\ str
    "value": \\ str
  }},
  ... \\ more fields
]
```
```

Extended Fig. 14: Prompt for the task of study characteristic extraction in all LLMs. Blue text indicates placeholders for variables within the prompt.

***Prompt for Arm Design Extraction***

```
# CONTEXT #
You are tasked with analyzing clinical trial study reports or papers to extract specific information as structured data.

# PAPER CONTENT #
{paper_content}

# TARGET #
Extract the arm design of the study.

# RESPONSE #
A syntactically correct JSON string:

Format:
```json
[ \\ a list of arms
    {{
    "label": \\ str, the arm label
    "type": \\ str, the arm type
    "description": \\ str, the description of the arm
    "interventionNames": \\ list of str, the interventions used in this arm
    }},
    {{
    "label": \\ str, the arm label
    "type": \\ str, the arm type
    "description": \\ str, the description of the arm
    "interventionNames": \\ list of str, the interventions used in this arm
    }},
    ... \\ more arms
]
```
```

Extended Fig. 15: Prompt for the task of arm design extraction in all LLMs. Blue text indicates placeholders for variables within the prompt.

***Prompt for Participant Statistics Extraction***

```
# CONTEXT #
You are tasked with analyzing clinical trial study reports or papers to extract specific information as structured data.

# PAPER CONTENT #
{paper_content}

# TARGET #
Given the following information:
Parameter Type: {paramType}
Unit of Measurement: {unitOfMeasure}
Participant Groups Definition: {groupDef}

Where:
"groupId" is the group identifier
"unit" is the unit of measurement for the group
"value" is the numerical value representing the group's characteristic
"def" is the definition or description of the group

Extract the target participant's characteristics about {measureDef}.

# RESPONSE #
A syntactically correct JSON string:

Format:
```json
{{
  "results": [ \\ the list of characteristics for the groups
    {{
      "groupId": \\ str
      "value": \\ float or int, the value for the group
      "note": \\ str, the note for the value, use simple words or phrases and do not use long sentences!
    }},
    ... \\ more results
  ]
}}
```
```

Extended Fig. 16: Prompt for participant statistics extraction in all LLMs. Blue text indicates placeholders for variables within the prompt.

***Prompt for Trial Result Extraction***

```
# CONTEXT #
You are tasked with analyzing clinical trial study reports or papers to extract specific information as structured data.

# PAPER CONTENT #
{paper_content}

# TARGET #
Extract the results related to the specified outcome and group as follows:

Outcome: {outcome_def}

Group: {group_def}

# RESPONSE #
A syntactically correct JSON string:

Format:
```json
{{
   "paramType": \\ str, the type of the parameters
   "unitOfMeasure": \\ str, the unit of the result values
   "timeFrame": \\ str, the timeframe
   "unitOfDenom": \\ str, the unit of the denomintor for this group
   "denomValue": \\ int, the value of the group's denominator
   "results": [ \\ list of result values
      {{
         "value": int or float \\ the result value
         "title": str \\ the title for this value
      }},... \\ more results, if applicable
   ]
}}
```
```

Extended Fig. 17: Prompt for the task of trial result extraction in all LLMs. Blue text indicates placeholders for variables within the prompt.

## ***Prompts for Search Query Generation in Baselines***

You are a clinical specialist. You are conducting research and doing a medical literature review.
The research is defined by the following PICO elements:
P (Patient, Problem or Population): {P}
I (Intervention): {I}
C (Comparison): {C}
O (Outcome): {O}

Your task is to create an URL of search query for relevant publications on PubMed.
template URL: https://eutils.ncbi.nlm.nih.gov/entrez/eutils/esearch.fcgi?db=pubmed&term=query+term
You should output an URL of search query based on it.

Your task is to create a URL for a search query to find relevant trials on ClinicalTrials.gov.
template URL: https://clinicaltrials.gov/api/v2/studies?query.term=query+term
You should output a URL for the search query based on this template.

One of objective for publication search or trial search

Notice:
1. Extract the most relevant and specific keywords from each PICO element.
2. Avoid using full sentences; focus on short, impactful terms.
3. Use Boolean operators (AND, OR) to structure your query logically.
4. Ensure the query is concise to maximize recall.
5. Use parentheses to group terms and control the query logic.
6. If there are synonymous terms or common variations, include them using the OR operator.

Steps to create the query:
1. Identify 1-2 primary keyword from each PICO element.
2. Combine these keywords using Boolean operators to form a structured search query.
3. Use parentheses to ensure proper grouping and logic in the query.
4. Include synonyms and variations using the OR operator to expand the search scope, if necessary.

Detailed Instruction or None

Your output should be in the following JSON format:
{{
"query": ”xxxxxx"
}}

Examples:

Example 1:
P: Adults with chronic hypertension
I: Treatment with ACE inhibitors
C: Comparison with beta-blockers and diuretics
O: Reduction in systolic blood pressure over 6 months
Query:
{{
"query": "https://eutils.ncbi.nlm.nih.gov/entrez/eutils/esearch.fcgi?db=pubmed&term=
chronic+hypertension+AND+ACE+inhibitors+AND+(beta-blockers+OR+diuretics)
+AND+systolic+blood+pressure+reduction"
}}

Example 2:
...

Example 3:
...

Provided Example or None

Current PICO:
P: {P}
I: {I}
C: {C}
O: {O}
Query:

Extended Fig. 18: Prompt for search query generation in baseline methods. Blue text indicates placeholders for variables within the prompt. The grey text represents optional sections of the prompt for different prompt types.

***Prompt for Study Eligibility Prediction in Simple Baselines***

```
Evaluate the following medical study paper against the provided PICO criteria.
Determine if this paper can be included in a literature review based on the PICO alignment.

Paper:
Title: {title}
Abstract: {abstract}

PICO Criteria:
Population (P): {P}
Intervention (I): {I}
Comparison (C): {C}
Outcome (O): {O}

Scoring Instructions:
Assign a score from 0 to 10 based on the alignment of the paper's title and abstract with the PICO criteria. (10: Perfect alignment, 0: No alignment)

Output Format:
Your output should be in the following JSON format:
```json
{{
  "score": 0-10
}}
```
```

Extended Fig. 19: Prompt for the task of study eligibility assessment in simple baseline methods. Blue text indicates placeholders for variables within the prompt.

## *Prompts for Study Eligibility Prediction in Advanced Baselines*

## *Stage 1: Criteria Generation*

````
Given the following PICO criteria for a medical literature review:
Population: {P}
Intervention: {I}
Comparison: {C}
Outcome: {O}

Generate {k} relevant evaluation criteria that can be used to assess the relevance of candidate papers based on these PICO criteria.
Each criterion should be clearly defined and focus on different aspects of the PICO elements.

Your output should be in the following JSON format:
{{
   "criteria": ["criteria_1", "criteria_2", ...]
}}
````

## *Stage 2: Evaluation*

````
Evaluate the following medical study paper against the provided criteria.
Determine if this paper can be included in a literature review based on the criteria alignment.

Paper:
Title: {title}
Abstract: {abstract}

Given the following criteria:
{criteria}

Scoring Instructions:
Assign a score from 0 to 10 based on the alignment of the paper's title and abstract with the given criteria. (10: Perfect alignment, 0: No alignment)

Output Format:
Your output should be in the following JSON format:
```json
{{
   "score": 0-10
}}
```
````

Extended Fig. 20: Prompt for the task of study eligibility assessment in advanced baseline methods. Blue text indicates placeholders for variables within the prompt.

***Prompt for GPT-4 Term Extraction***

```
# CONTEXT #
You are a clinical specialist. You are conducting research and doing a medical literature review.

# OBJECTIVE #
Your task is to extract PICO medical terms from a medical literature.
P: Patient, Problem, or Population
I: Intervention
C: Comparison
O: Outcome

# LITERATURE DETAILS #
Title: {title}
Abstract: {abstract}

# NOTICE #
You should first extract basic medical terms from the title and abstract.
Each basic medical term should be concise and representative, avoiding general terms.
Do not include general medical terms, such as intervention, trial, study, analysis, adolescent, adult, female, male, humans, mice,
survey, questionnaire, efficacy, etc., or any non-representative terms.
Then you should extract 1-3 important PICO terms for each PICO element from the basic terms.
Ensure that PICO terms do not include subject information like adult, male, etc.; only include descriptive words and subject
characteristics in P terms.

# RESPONSE #
A syntactically correct JSON string:

Format:
'''json
{{
    "basic_terms": ["term1", "term2", ...],
    "p_terms": ["term1", "term2", ...],
    "i_terms": ["term1", "term2", ...],
    "c_terms": ["term1", "term2", ...],
    "o_terms": ["term1", "term2", ...],
}}
'''
```

Extended Fig. 21: Prompt used to extract medical terms from a study by GPT-4. Blue text indicates placeholders for variables within the prompt.

***Prompt for GPT-4 Study Eligibility Analysis***

```
# CONTEXT #
You are a clinical specialist tasked with assessing research papers for inclusion in a systematic literature review based on specific
eligibility criteria.

# OBJECTIVE #
You will be given a paper which was [involved / not involved]  in the target systematic review judged by a human expert before.
It means that the paper generally [meets / does not meet] all the criteria. But it does not mean that the paper [is eligible for all / not
eligible for any of] the specified criteria.
Analyze the reason this paper [can /cannot] be included in the systematic review and provide an explanation.
Evaluate each criterion of a given paper to determine its eligibility for inclusion in the review. Provide a list of decisions ("YES",
"PARTIAL", "NO", or "UNCERTAIN") for each eligibility criterion. You must deliver exactly {num_criteria} responses.
1. YES: Meets the criteria.
2. PARTIAL: Partially meets the criteria but not completely.
3. NO: Does not meet the criteria.
4. UNCERTAIN: Uncertain if it meets the criteria.

# IMPORTANT NOTE #
If the information within the provided paper content is insufficient to conclusively evaluate a criterion, you must opt for "UNCERTAIN"
as your response. Avoid making assumptions or extrapolating beyond the provided data, as accurate and reliable responses are crucial,
and fabricating information (hallucinations) could lead to serious errors in the systematic review.
If the information is not applicable N/A, you also must opt for "UNCERTAIN".
Use "PARTIAL" when the paper meets some aspects of the criterion but not all; ensure that the partial fulfillment is based on the
provided data and not on assumptions or incomplete information.

# PAPER DETAILS #
- Provided Paper that was involved in the target systematic review: {paper_content}

# EVALUATION CRITERIA #
- Number of Criteria: {num_criteria}
- Criteria for Inclusion: {criteria_text}

# RESPONSE #
You are required to output a JSON object containing a list of decisions for each of the {num_criteria} eligibility criteria. Each decision
should directly correspond to one of the criteria and be listed in the order they are presented. Ensure to use "UNCERTAIN" wherever
the paper does not explicitly support a "YES", "PARTIAL", or "NO" decision.
The length of "evaluation" should be exactly {num_criteria}.
For example:
```json
{{
  "explanation": "...", \\ one sentence explaining why this paper can be included in the systematic review
  "evaluations": [ \\ list of eligibility decisions for each criterion
    {{
      "eligibility": "YES", \\ decision for the first criterion
      "rationale": "..." \\ rationale for the decision, limited in 50 tokens
    }},
    {{
      "eligibility": "PARTIAL", \\ decision for the second criterion
      "rationale": "..." \\ rationale for the decision, limited in 50 tokens
    }},
    {{
      "eligibility": "NO", \\ decision for the third criterion
      "rationale": "..." \\ rationale for the decision, limited in 50 tokens
    }},
    {{
      "eligibility": "UNCERTAIN", \\ decision for the fourth criterion
      "rationale": "..." \\ rationale for the decision, limited in 50 tokens
    }},
    ... \\ continue for all criteria
  ]
}}
```
```

Extended Fig. 22: Prompt used to analyze the eligibility reasons for a study based on specified criteria by GPT-4. Blue text indicates placeholders for variables within the prompt. Red text changes depending on whether the current study is eligible or not.
