# Supplementary material for: A foundation model for human-AI collaboration in medical literature mining
Source: Nat Commun. 2025 Sep 24;16:8361. doi: 10.1038/s41467-025-62058-5 (PMC12460617; doi:10.1038/s41467-025-62058-5)
Supplement: Supplementary file 2 — Reporting Summary [file 41467_2025_62058_MOESM2_ESM.pdf]

nature portfolio

Corresponding author(s): Zifeng Wang, Jimeng Sun

Last updated by author(s): Jun 12, 2025

Reporting Summary

Nature Portfolio wishes to improve the reproducibility of the work that we publish. This form provides structure for consistency and transparency in reporting. For further information on Nature Portfolio policies, see our Editorial Policies and the Editorial Policy Checklist.

Statistics

For all statistical analyses, confirm that the following items are present in the figure legend, table legend, main text, or Methods section.

n/a	Confirmed	
☐	☒	The exact sample size (*n*) for each experimental group/condition, given as a discrete number and unit of measurement
☐	☒	A statement on whether measurements were taken from distinct samples or whether the same sample was measured repeatedly
☐	☒	The statistical test(s) used AND whether they are one- or two-sided<br>*Only common tests should be described solely by name; describe more complex techniques in the Methods section.*
☐	☒	A description of all covariates tested
☐	☒	A description of any assumptions or corrections, such as tests of normality and adjustment for multiple comparisons
☐	☒	A full description of the statistical parameters including central tendency (e.g. means) or other basic estimates (e.g. regression coefficient) AND variation (e.g. standard deviation) or associated estimates of uncertainty (e.g. confidence intervals)
☐	☒	For null hypothesis testing, the test statistic (e.g. *F*, *t*, *r*) with confidence intervals, effect sizes, degrees of freedom and *P* value noted<br>*Give P values as exact values whenever suitable.*
☒	☐	For Bayesian analysis, information on the choice of priors and Markov chain Monte Carlo settings
☒	☐	For hierarchical and complex designs, identification of the appropriate level for tests and full reporting of outcomes
☐	☒	Estimates of effect sizes (e.g. Cohen's *d*, Pearson's *r*), indicating how they were calculated

Our web collection on statistics for biologists contains articles on many of the points above.

Software and code

Policy information about availability of computer code

Data collection	Data were collected from PubMed via the PubMed FTP service, publicly available at https://pubmed.ncbi.nlm.nih.gov/download/ and https://pmc.ncbi.nlm.nih.gov/tools/ftp/. Clinical trial data were obtained from ClinicalTrials.gov using the AACT database, which is publicly accessible at https://aact.ctti-clinicaltrials.org/downloads.
Data analysis	All experiments were run in Python 3.12. Detailed software versions are: vLLM v0.6.4.post1, openai v1.55.1, transformers v4.46.3, pytorch v2.5.1. Source code can be accessed at https://github.com/RyanWangZf/LEADS.

For manuscripts utilizing custom algorithms or software that are central to the research but not yet described in published literature, software must be made available to editors and reviewers. We strongly encourage code deposition in a community repository (e.g. GitHub). See the Nature Portfolio guidelines for submitting code & software for further information.

Data

Policy information about availability of data

All manuscripts must include a data availability statement. This statement should provide the following information, where applicable:

- Accession codes, unique identifiers, or web links for publicly available datasets
- A description of any restrictions on data availability
- For clinical datasets or third party data, please ensure that the statement adheres to our policy

The PubMed publication data are publicly available at https://pubmed.ncbi.nlm.nih.gov/download/. The PubMed Central publication data are publicly available at

https://pmc.ncbi.nlm.nih.gov/tools/ftp/. The clinical trial records are publicly available at https://aact.ctti-clinicaltrials.org/downloads. The LEADSInstruct data generated in this study have been deposited in the Hugging Face database under accession code: https://huggingface.co/datasets/zifeng-ai/LEADSInstruct, including training, testing, and development datasets for search query generation, study eligibility evaluation, study characteristic extraction, trial result extraction, participant statistics extraction, and arm design extraction. Source data are provided with this paper.

Research involving human participants, their data, or biological material

Policy information about studies with human participants or human data. See also policy information about sex, gender (identity/presentation), and sexual orientation and race, ethnicity and racism.

Reporting on sex and gender	No human data is involved in this study.
Reporting on race, ethnicity, or other socially relevant groupings	No human data is involved in this study.
Population characteristics	No human data is involved in this study.
Recruitment	No human data is involved in this study.
Ethics oversight	No human data is involved in this study.

Note that full information on the approval of the study protocol must also be provided in the manuscript.

Field-specific reporting

Please select the one below that is the best fit for your research. If you are not sure, read the appropriate sections before making your selection.

☒ Life sciences ☐ Behavioural & social sciences ☐ Ecological, evolutionary & environmental sciences

For a reference copy of the document with all sections, see nature.com/documents/nr-reporting-summary-flat.pdf

Life sciences study design

All studies must disclose on these points even when the disclosure is negative.

Sample size	633,759 instruction data points, curated from 21,335 systematic reviews, 453,625 clinical trial publications, and 27,015 clinical trial registries are involved. No statistical methods were used to predetermine the sample size. The sample size was then determined by data acquisition, followed by a data exclusion and filtering pipeline. The large data size yields statistically significant experiment results.
Data exclusions	We removed duplicates, citations lacking essential information, and reviews without associated citations
Replication	n/a. Replications are not applicable because the computational results are deterministic without randomness.
Randomization	n/a; not an interventional study
Blinding	n/a; not an interventional study

Reporting for specific materials, systems and methods

We require information from authors about some types of materials, experimental systems and methods used in many studies. Here, indicate whether each material, system or method listed is relevant to your study. If you are not sure if a list item applies to your research, read the appropriate section before selecting a response.

Materials & experimental systems

n/a	Involved in the study	
☒	☐	Antibodies
☒	☐	Eukaryotic cell lines
☒	☐	Palaeontology and archaeology
☒	☐	Animals and other organisms
☒	☐	Clinical data
☒	☐	Dual use research of concern
☒	☐	Plants

Methods

n/a	Involved in the study	
☒	☐	ChIP-seq
☒	☐	Flow cytometry
☒	☐	MRI-based neuroimaging

Plants

Seed stocks	n/a
Novel plant genotypes	n/a
Authentication	n/a
